# Widespread Triploidy in Western North American Aspen (*Populus tremuloides*)

**DOI:** 10.1371/journal.pone.0048406

**Published:** 2012-10-31

**Authors:** Karen E. Mock, Colin M. Callahan, M. Nurul Islam-Faridi, John D. Shaw, Hardeep S. Rai, Stewart C. Sanderson, Carol A. Rowe, Ronald J. Ryel, Michael D. Madritch, Richard S. Gardner, Paul G. Wolf

**Affiliations:** 1 Department of Wildland Resources, Utah State University, Logan, Utah, United States of America; 2 United States Department of Agriculture Forest Service, Southern Institute of Forest Genetics, Forest Tree Molecular Cytogenetics Laboratory, Texas A & M University, College Station, Texas, United States of America; 3 United States Department of Agriculture Forest Service, Rocky Mountain Research Station, Ogden, Utah, United States of America; 4 United States Department of Agriculture Forest Service, Rocky Mountain Research Station, Shrub Sciences Laboratory, Provo, Utah, United States of America; 5 Department of Biology, Appalachian State University, Boone, North Carolina, United States of America; 6 Department of Biology, Utah State University, Logan, Utah, United States of America; United States Department of Agriculture, United States of America

## Abstract

We document high rates of triploidy in aspen (*Populus tremuloides*) across the western USA (up to 69% of genets), and ask whether the incidence of triploidy across the species range corresponds with latitude, glacial history (as has been documented in other species), climate, or regional variance in clone size. Using a combination of microsatellite genotyping, flow cytometry, and cytology, we demonstrate that triploidy is highest in unglaciated, drought-prone regions of North America, where the largest clone sizes have been reported for this species. While we cannot completely rule out a low incidence of undetected aneuploidy, tetraploidy or duplicated loci, our evidence suggests that these phenomena are unlikely to be significant contributors to our observed patterns. We suggest that the distribution of triploid aspen is due to a positive synergy between triploidy and ecological factors driving clonality. Although triploids are expected to have low fertility, they are hypothesized to be an evolutionary link to sexual tetraploidy. Thus, interactions between clonality and polyploidy may be a broadly important component of geographic speciation patterns in perennial plants. Further, cytotypes are expected to show physiological and structural differences which may influence susceptibility to ecological factors such as drought, and we suggest that cytotype may be a significant and previously overlooked factor in recent patterns of high aspen mortality in the southwestern portion of the species range. Finally, triploidy should be carefully considered as a source of variance in genomic and ecological studies of aspen, particularly in western U.S. landscapes.

## Introduction

Species distributions are the manifestation of complex evolutionary and ecological histories. Understanding the drivers of species distribution has long been a central theme in ecological research, and has taken on new urgency as society struggles to predict and mitigate the impacts of rapid climate change. Evolutionary and ecological effects are often nested, such that the distribution and diversity of a foundation species becomes a matrix influencing the distribution and diversity of dependent species [1]. Therefore, understanding the factors driving the distribution of widespread, foundation species can be insightful in understanding and predicting distributions of associated species and communities.

One of the intrinsic factors influencing plant distributions is clonality. Most plants are capable of some degree of both clonality and sexuality, and each reproductive mode is associated with particular advantages and costs [2]–[4]. Clonal size often increases with latitude [5], elevation [6]–[8], and position at range edges [9], and is thought to allow persistence in harsh environments where sexual recruitment may be limited or episodic [10]–[12]. In some environments, clonal persistence can be dramatic, on the order of millennia [13]–[15].

Another intrinsic factor potentially influencing species distribution is polyploidy. Individuals or lineages with different cytotypes (e.g. diploid, tetraploid) typically occupy distinct geographic and ecological space [16], [17]. The presence of three or more copies of the genome can have various physiological effects, and may increase longevity due to heterosis, mutational buffering, or altered rates of growth [16], [18], [19]. An assortment of hypotheses has addressed the geographic distribution of polyploidy, including a preponderance of polyploid forms in cold climates, post-glacial habitats, and high elevations or latitudes [18], [20]–[24]. The most direct assessments of the ecological impacts and drivers of polyploidy can be made when cytotypes vary within natural populations. However, cytotypes with an even number of chromosome sets can form sexually reproducing lineages that follow independent evolutionary trajectories and histories, and lineages with different cytotypes may not be sympatric.

**Figure 1:**
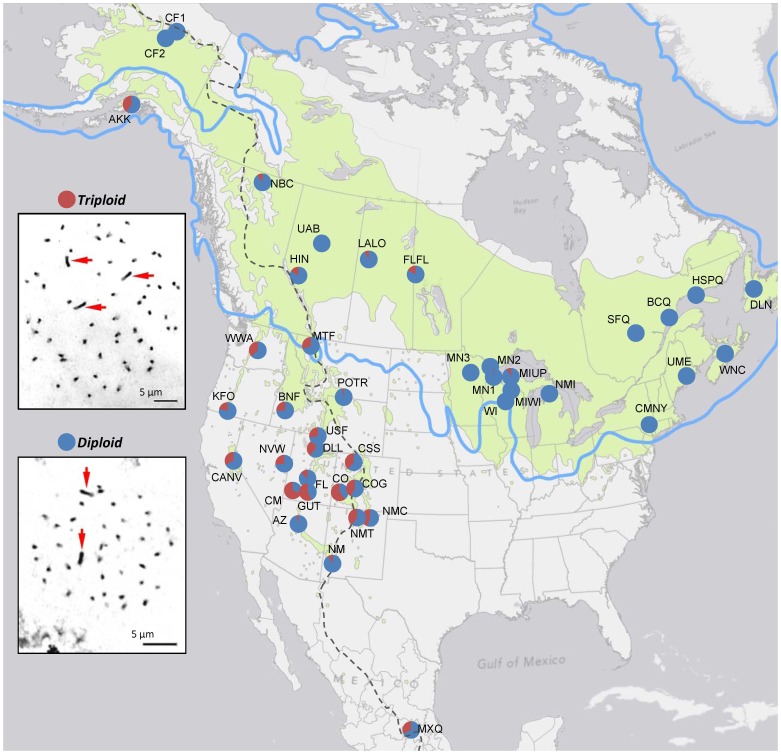
Rangewide cytotype proportions in *Populus tremuloides*. Current species range (green) [59] continental divide (dashes), and last glacial maximum (blue line) [58] are shown. Insets show somatic metaphase chromosome spreads (diploid, 2*n* = 38; triploid, 3*n* = 57), red arrow indicates chromosome 1, the largest in the genome.

Polyploidy in natural plant populations generally results from fertilization with unreduced gametes (from 2n spores in diploids) [25], [26]. The rate of unreduced spore production in meiosis may be influenced by genetic or environmental variables, and can be elevated in hybrids [22], [26], [27]. In the case of autopolyploidy, triploids may play an important role in the generation of sexual tetraploids [22]. In diploid nonhybrid populations, autotriploids have been observed in many species but occur at low frequencies (e.g. [28]–[30]). Such triploids have reduced fertility but may persist vegetatively, particularly when environmental circumstances reduce intraspecific competition. Additionally, selection against the formation of unreduced spores may be relaxed in long-lived perennials [22]. Thus, ecological factors that promote clonality may also promote both the incidence and persistence of triploids, particularly when triploids have vegetative advantages [22], [31]. Given this potential synergy, the frequency of triploid genets may be highest in long-lived, clonal perennials [32]. Further, the incidence of clonality and triploidy might be expected to co-occur when they both vary within a species, and these traits could contribute to the ecological amplitude and geographic range of species distributions. Alternatively, if triploidy (or the production of unreduced spores) and clonality are responding to different ecological drivers, their landscape variances could be distinct.

**Table 1:** Microsatellite locus sets used for genotyping.

µsat locus	Set 1	Set 2	Set 3	Set 4	Set 5	Set 6	Set 7
PMGC-2571	X	X	X	X	X	X	X
PMGC-2658	X			X			X
PMGC-486	X			X			X
PMGC-510	X			X			X
WPMS-14	X	X	X	X	X	X	X
WPMS-15	X	X	X	X	X	X	X
WPMS-17	X			X	X		X
WPMS-20	X	X	X	X			X
GCPM-2768					X		
GCPM-970		X	X		X	X	X
PMGC-433		X	X		X	X	
PMGC-576		X	X	X			X
PTR-14			X		X	X	
WPMS-16			X				
**Total**	**8**	**7**	**9**	**9**	**8**	**6**	**10**

Microsatellite (µsat) primer sequences are available as follows: PMGC and GCPM (http://www.ornl.gov/sci/ipgc/ssr_resource.htm); WPMS [76]; PTR [77].

In this study we characterize the geographic distribution of triploidy in a widespread, long-lived clonal species, trembling aspen (*Populus tremuloides*; hereafter ‘aspen’) relative to broad patterns of clonality, glaciation, and climate. Aspen is an ideal species for addressing questions about continental-scale variation in clonality and ploidy because of its extensive geographic/climatological range, its tendency to produce large clones in certain geographic regions, and the recent finding of putative triploids in high frequencies in local surveys. Our findings have implications for aspen ecology as well as the evolutionary ecology of highly clonal plant species.

## Materials and Methods

### Study Species

Aspen is among the most important forest species in North America. The geographic and ecological amplitude of aspen is immense, spanning over 111° of longitude and 48° of latitude, and occupying elevations ranging from sea level to over 3500 m [33]–[35]. Aspen is the most broadly distributed tree in North America, and the most common broadleaf tree in Canada’s immense boreal forest [36]. Aspen has tremendous ecological, economic, and aesthetic value, particularly in the western portion of its range, where it is associated with disproportionately high biodiversity relative to other forest trees [37]. High rates of mortality have been reported for western aspen in recent years [38]–[40], attributable to various proximal causes, including pathogens, succession, herbivory, and water stress [41], [42], and potentially resulting in significant pulses of carbon emission [43]. Bioclimatic models predict rapid transitions to more arid climates in the western U.S. [44], with habitat losses of up to 94% for aspen in western landscapes within a century [45]. Accurate forecasting, mitigation, and restoration will require an integrated understanding of factors influencing aspen distribution and regeneration.

**Figure 2:**
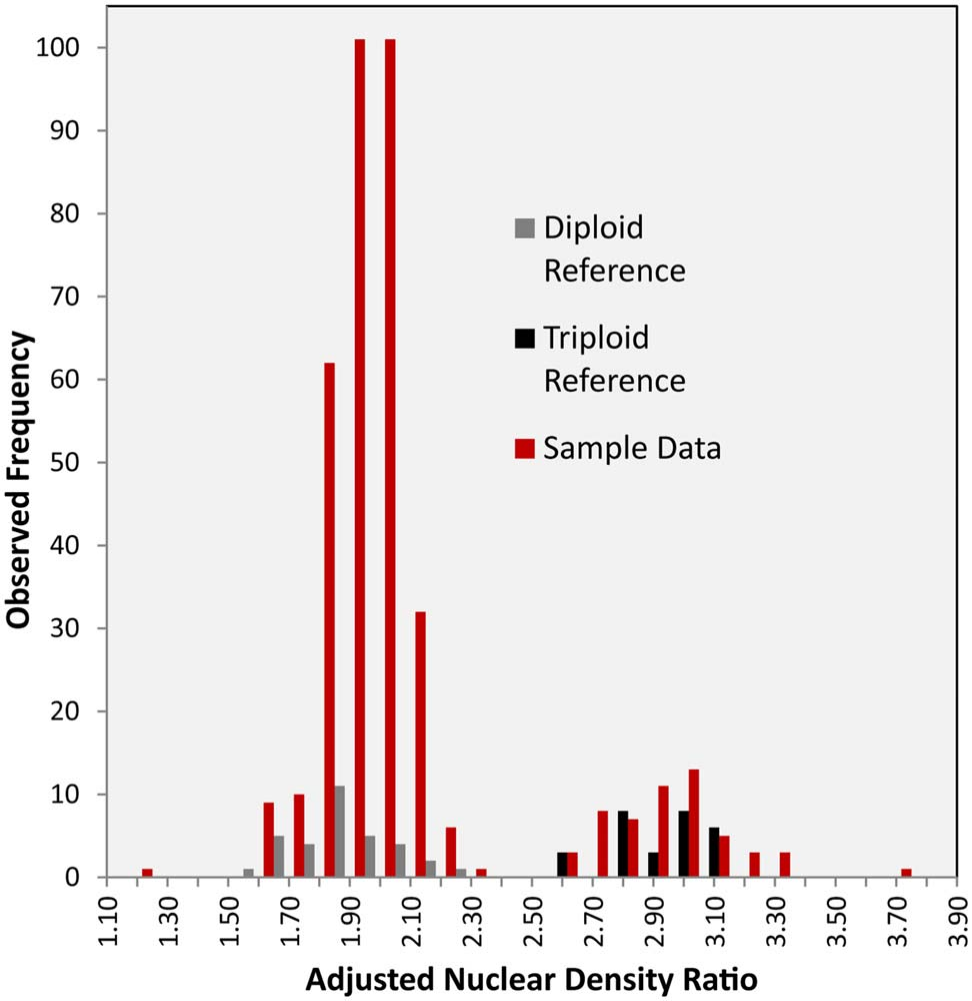
Frequency distribution of nuclear DAPI staining intensity ratios measured by flow cytometry. Diploid and triploid reference samples from 2 individuals were included in all sets of analyses; variance within these individuals represents technical error. Sample data represents rangewide dataset.

**Figure 3:**
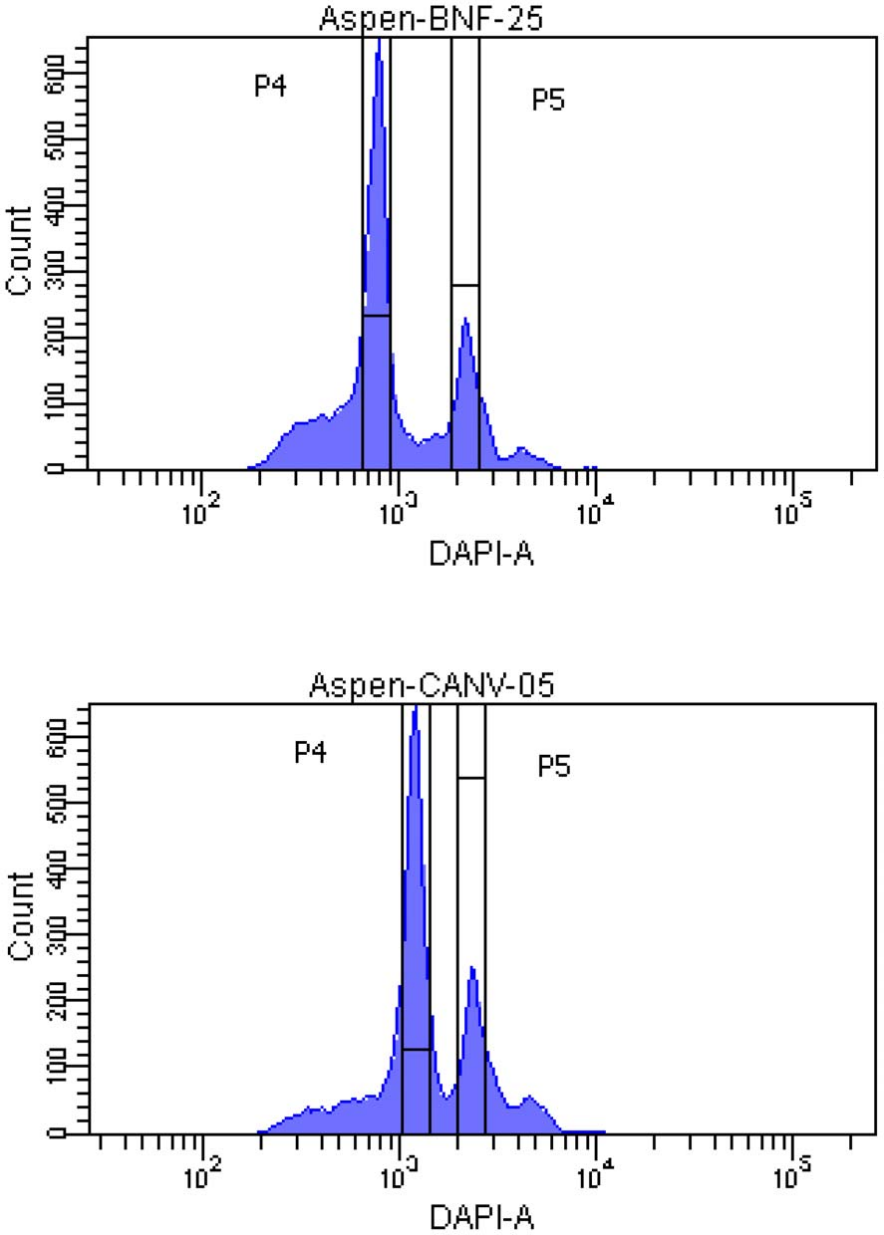
Flow cytometry panels from a representative diploid (BNF_25; upper panel) and triploid (CANV_05; lower panel). In both panels, P4 is aspen and P5 is the reference sample (*Atriplex canescens*). DAPI-A indicates the area (A) of the DAPI (nuclear stain) fluorescence emitted by each nucleus. The vertical axis shows the count of the number of nuclei in each fluorescence channel. Vertical bars enclose the range of data used to calculate median fluorescence for each peak.

Aspen is perhaps the plant species most renowned for its clonality; the largest living organism documented to date is an aspen clone (“Pando”) occupying 43.3 ha in central Utah [46], [47]. Clone (genet) size in aspen varies dramatically across the continent: east of the Rocky Mountains, clones are typically under 0.04 ha, but in the Rocky Mountains, clones can cover many hectares [46], [48]. The continent-scale variance in aspen clone size is putatively determined by the frequency and density of seedling establishment, regional disturbance regimes, and competition with more shade-tolerant species [46], consistent with our general understanding of clonal advantages. In landscapes of the Rocky Mountains and Intermountain West, seedling establishment is limited by xeric conditions and tends to be episodic following disturbances that leave unshaded mineral soils with adequate moisture (e.g. following fires) [49].

**Figure 4:**
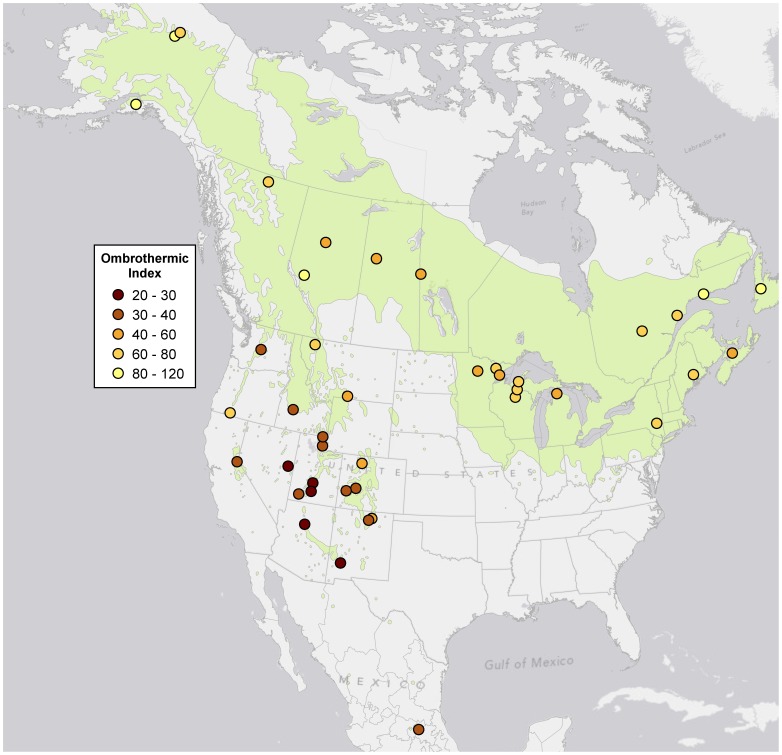
Ombrothermic index for aspen sampling sites, based on North American Regional Reanalysis (NARR) data from 1990–2010. NARR data are mapped on a 32-km resolution grid, so values assigned to each sampling site are for the grid cell intersected by the sample cluster centroid.

**Figure 5:**
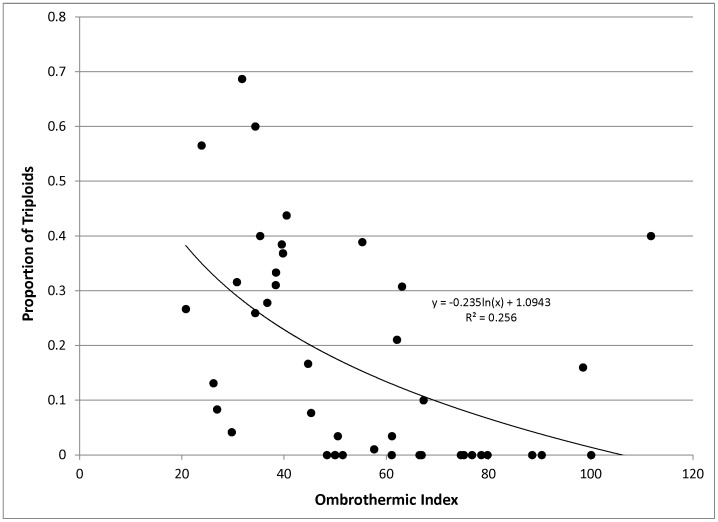
Regression between ombrothermic index and proportion of triploids. Data points represent the ploidy proportions mapped in Figure 1 and the ombrothermic index values mapped in Figure 4.

Local western stand surveys have recently revealed a high proportion of aspen genets with three microsatellite alleles per locus, suggesting triploidy [50]. These same surveys suggested that the larger clones were triploids. Previous studies have documented triploidy in aspen, both in the eastern and western portions of the range [51], [52], and triploid individuals are of interest in silviculture because of their desirable growth and fiber properties [51], [53]. However, the frequency of naturally occurring triploids in aspen has not been investigated at a large spatial scale. Further, triple alleles at microsatellite loci may also be the result of aneuploidy, segmental duplication, or homeology. The latter is a particular possibility in aspen, as poplars have undergone ancient genome duplication [54], [55].

**Table 2:** Microsatellite set 1 summary data.

	Locus							
	WPMS-14	WPMS-15	WPMS-17	WPMS-20	PMGC-486	PMGC- 510	PMGC- 2571	PMGC- 2658
Total number of alleles observed	22	11	14	13	33	30	28	24
Frequency of three most commonly observed alleles	0.3060.2620.117	0.3750.1670.126	0.5660.1340.072	0.5810.1370.088	0.2320.1200.105	0.1340.1050.079	0.1540.1180.095	0.1490.1100.092
Maximum number of alleles observed within a sampling site	11	9	10	7	22	21	22	20
Proportion of genets with triple alleles at P486 which hadtriple alleles at each other locus	0.355	0.563	0.219	0.097	n/a	0.625	0.469	0.355
Proportion of genets without triple alleles at P486 which had triple alleles at each other locus	0.025	0.018	0.012	0.005	n/a	0.031	0.029	0.041
Ratio (B:A)	1∶14.2	1∶31.3	1∶18.2	1∶19.4	n/a	1∶20.2	1∶16.2	1∶8.7

Information on loci and sampling sites provided in Tables 1 and S1.

### Sample Acquisition and Ethics Statement

Mid-season leaves were collected from georeferenced trees dispersed across 42 sampling sites of varying sizes; maximum distances between trees within a site ranged from 1 to 97 km (Figure 1, Table S1). Leaf samples were dried and stored in silica desiccant. Cytotypes were assessed using microsatellite genotypes (6–10 unlinked loci; 1147 genets), flow cytometry (139 genets), or both (296 genets), for a total of 1582 sampled genets. Leaf sample collections were made on public lands with appropriate permissions and notifications (US Forest Service), collected and provided by representatives of federal or regional agencies, or provided by academic researchers in conjunction with other studies where such permissions were obtained.

### Microsatellite Analysis

Genomic DNA was extracted from dried leaf tissue using a Qiagen DNeasy 96 Plant kit. Sets of between 6−10 unlinked microsatellite loci (Tables 1 & S1) were used to identify individual genets. Although data from various microsatellite panels were assembled in this study, all DNA extractions and microsatellite analyses were performed in the same laboratory using appropriate positive and negative controls. PCR reactions followed standard protocols [50], and PCR products were multiplexed in pairs prior to fragment analysis using an ABI 3730 automated DNA Sequencer with a LIZ500 size standard. Duplicate samples from identical genets were excluded from our analyses.

Triple alleles in an individual could be due to duplicated loci (e.g. from homeologous loci [54], aneuploidy, or segmental duplication), genotyping error, or higher level ploidy, rather than triploidy. In the case of duplicated loci, we would expect to observe four-allele genotypes at loci with high allelic diversity, and that priming site duplication would be a locus-specific phenomenon, not a genome-wide phenomenon. Similarly, the effect of genotyping error is expected to be locus-specific. In order to assess locus independence with respect to triple allele incidence, we determined whether individuals with three alleles at the most variable locus (PMGC-486) had an elevated incidence of three alleles at other loci, as would be expected with triploidy but not with alternative explanations. We made this assessment in a set of 810 individuals using the most common combination of microsatellite loci (Set 1, Table 1). Based on our findings (see Results), we designated individuals as triploids if they had three alleles at any locus. Flow cytometry was used to confirm these results and to assess the incidence of other cytotypes (see below).

Naturally occurring triploids may be the result of interspecific hybridization [22], [26]. Although aspen hybridizes naturally with other *Populus* species, hybridization is not common except where *P. tremuloides* and *P. grandidentata* co-occur. Nonetheless, to exclude hybridization as a source of elevated triploidy, we compared allele frequencies between diploid and triploid genets in the three largest sample sets containing both cytotypes (Table S1: sites CM, USF, and BNF), expecting that allotriploids would have different allele frequencies than non-hybridized diploids.

### Flow Cytometry

We performed flow cytometry on 435 samples from across the species range; 296 of these samples were also genotyped, allowing assessment of cytotype consistency between microsatellite and flow cytometry results (Table S1). When choosing genotyped samples for flow cytometry, we made an effort to include a disproportionate number of samples which had been deemed triploid based on a single microsatellite locus, expecting that these samples would be at greatest risk for misclassification due to genotyping error. Dried aspen leaf fragments (15–20 g) were mixed with 25 g of dried *Atriplex canescens* leaf tissue (a reference sample) in 2-mL microcentrifuge tubes. With every run of up to 24 samples, we also included samples of two reference aspen trees, one diploid and one triploid (defined by both microsatellite alleles and flow cytometry), to assess technical error (Figure 2). Leaf fragments were suspended in 500 µL CyStain® UV Ploidy staining solution and pulverized using single ceramic beads (1/4-inch spheres, MP Biomedicals) with a Qiagen Retsch MM300TissueLyser. The suspension was filtered using Partec CellTrics disposable tube top filters, and an additional 1150 µL of CyStain® was added. Filtrates were analyzed on a BD Biosciences SORP FACSAria II equipped with four lasers: a 40 mW Red 640 nm laser, a 100 mW Green 561 nm laser, a 100 mW Blue 488 nm laser, and a 60 mW Ultraviolet (UV) 355 nm laser. The DAPI nuclear stain was excited by the UV laser and the emission signal was isolated using a 450/50 bandpass filter. DIVA software was used for data acquisition and analysis. Samples were gated on forward and side scatter to filter out cellular debris and clumps of nuclei. The remaining events were plotted on a two dimensional plot with the area of the DAPI fluorescence on the X axis and the number of events per channel on the Y axis. Gates were manually set around the sample (aspen) and reference (*A. canescens*) peaks. The ratio of the mean fluorescence for the two peaks was used to determine the ploidy of the aspen sample (Figure 3).

### Cytology

Root segments of approximately 1 mm diameter were field-collected from one putative triploid and three putative diploids genets (based on microsatellite genotypes and flow cytometry) from Utah and Arizona. Shoots were propagated in a greenhouse setting [56]. Actively growing root tips about 1 cm long were excised and pretreated with a saturated aqueous solution of α-bromonapthalene (0.8%) for 2 h in the dark at room temperature and then fixed in 4∶1 (95% ethanol – glacial acetic acid) solution. The root tips were processed enzymatically and the chromosome spreads were prepared [57]. The preparation was then stained with 0.2% Azure Blue (Sigma) and made permanent with a drop of Euparol. The chromosome spreads were viewed under a 63X Plan Apo-chromatic objective using AxioImager M2 microscope (Carl Zeiss, Germany) and digital images were recorded and pre-processed using ISIS v5.1 (MetaSystem Group Inc.). Multiple spreads from each of the four individuals were prepared and examined.

### Mapping

Proportions of triploid and diploid genets for each sampling site were projected onto a map with respect to the continental divide, the last glacial maximum [58], and the species range [59] using ArcMap10 (ESRI, California, USA) (Figure 1). For each site we also computed an ombrothermic index, using a 21-year dataset (1990–2010) from the North American Regional Reanalysis project [60] (ftp://nomads.ncdc.noaa.gov/NARR_monthly/accessed November 1, 2011) The ombrothermic index (OI) was calculated as (PSUM/TSUM)10, where PSUM and TSUM are the total precipitation (mm) and the sum of mean temperature (°C), respectively, over months with a mean temperature of over 0°C.

## Results

Proportions of triploid genets within sampling sites ranged from 0–69%. Triploidy was highest in portions of the range west of the continental divide, south of the last glacial maximum, and particularly high in southern Utah and western Colorado (Figure 1). A correspondence between triploidy and climate (OI) was evident both visually (Figure 4) and via a regression analysis (Figure 5), although there was significant scatter in the data, potentially due to the coarse scale of both the OI mapping and the sample collection.

Several lines of evidence suggested that the triple alleles observed in microsatellite genotypes were due to triploidy and not duplicated loci or tetraploidy. Individuals with triple alleles were observed at each of the loci assayed, and we never observed an individual with four alleles at a locus, despite high allelic richness across loci (Table 2). With microsatellite Set 1, individuals with three alleles at the most variable locus (PMGC-486) had an elevated incidence of triple alleles at all other loci, generally an order of magnitude greater than the incidence in individuals which had two alleles at the most variable locus (Table 2). Within individuals, triple alleles were observed at an average of 31% of loci genotyped.

Consistency between microsatellite genotyping and flow cytometry results was high when both were analyzed; we found cytotype inconsistencies in 6/296 (2.02%) samples (4 had three microsatellite alleles but were diploid by flow cytometry; 2 were the reverse). We failed to detect any tetraploids using flow cytometry. Chromosome counts in three diploid individuals and one triploid individual confirmed the diploid (2*n* = 38) and triploid (3*n* = 57) cytotypes suggested by microsatellite and flow cytometric analyses (Figure 1). Aneuploidy in some cases cannot be entirely ruled out, although the distribution of nuclear densities in flow cytometry was strongly bimodal (Figure 2).

Results of allele frequency comparisons between pools of diploid and triploid individuals within populations were consistent with autotriploidy resulting from unreduced spore formation, and suggested that triploidy was not due to hybridization. The most commonly observed alleles in populations CM, USF, and BNF were identical between diploids and triploids, but distinct among populations, and we found no evidence of high frequency (>0.15) alleles unique to either triploids or diploids.

Because of our use of microsatellite genotypes to identify most cytotypes, we cannot completely rule out the possibility of undetected tetraploids among our samples. However, it is unlikely that these made up a substantial portion of our samples, given the failure to detect genotypes with four alleles at a locus despite high allelic diversity within populations and the failure to detect tetraploid nuclei using flow cytometry.

## Discussion

Our continent-wide survey, based on 1582 genets from 42 sites, revealed that triploids represent a significant proportion of genets in this important forest species. Triploidy is most common in the southwestern portion of the species range (Figure 1), where seedling survival is expected to be rare and where the ecological value and vulnerability of the species are particularly high [45], [61]. Previous instances of triploidy in aspen have been documented by limited cytological observations [51]–[53], but a recent study using microsatellite genotyping [50] suggested that the frequency of triploidy might be quite high in some landscapes. In this study, over half of the genets in some Colorado and Utah sites were triploids, and triploidy generally corresponded with the geography of large clone sizes [49]. These results are consistent with a hypothesized synergy between clonality and triploidy, and the elevated frequency of these traits in more drought-prone regions suggests that these factors may extend the ecological amplitude and geographic range of the species. Given the tendency for larger aspen clones to be triploid [50], the spatial area occupied by triploid clones in western landscapes could far exceed that occupied by diploid clones.

Continental patterns of triploidy did not correspond with the latitudinal gradients or recent glacial histories often associated with polyploidy [23]. In fact, the highest incidences of triploidy occurred in unglaciated portions of the range (Figure 1). Ombrothermic index (OI) explained a significant portion of the continental-scale variability in triploidy (Figures 4 and 5), suggesting a role for climate.

There are at least three potential explanations for this continental pattern: 1) ecological drivers in the western portion of the species range may favor the formation of unreduced spores, 2) western populations may comprise a distinct lineage(s) with a heritable tendency to produce unreduced spores, and/or 3) ecological drivers favoring clonality may also favor triploid genets over diploid genets. These explanations need not be mutually exclusive.

Regarding the first explanation, the rate of unreduced spore formation has not been directly examined in aspen, but evidence in other plant species suggests that this rate may be increased by a number of environmental stressors (climatic, nutritional, and herbivore- or disease-related factors) [19]. Continental patterns in these stressors could influence triploid incidence in aspen, particularly if arid climates are a driver of unreduced spore formation. Evidence also exists for the second scenario: a recent rangewide study indicates that aspen in the southwestern portion of the species range represent a distinct genetic cluster from aspen in the northern portion of the range, based on nuclear microsatellite allele frequencies [62]. Although the geographic range of the southwestern genetic cluster does not extend to Alaska, where rates of triploidy were also high (AKK; Figure1), there is a strong general correspondence between triploid rates and intraspecific genetic structuring.

Under the third scenario, if triploids enjoy an advantage over diploids with respect to vegetative growth (clonal expansion rates and/or ramet growth) or persistence, they could be favored in environments favoring long-lived genets. Given that early growth of aspen from seed is highly sensitive to temperature and moisture [63], clonal growth would be advantageous in areas with warmer, drier summers (i.e. lower OI values). If triploids have a vegetative advantage over diploids, then triploid genets would be more common and larger in areas with a low OI. Data from previous local studies support this explanation: genets covering larger areas tend to be triploid [50] (including the enormous “Pando” clone [64]), suggesting a strong vegetative advantage for triploids regardless of the rate of their formation. The incidence of triploidy also generally corresponds with the potential for clonal antiquity, if clonal ages frequently exceed the age of the last glacial maximum (19–26,000 years) [15], [65]. Additionally, one of our surveyed areas (Table S1: AZ) consisted of samples from seedlings emerging after a high-intensity fire in 2000. This location was associated with the lowest rate of triploidy found west of the continental divide, suggesting that triploid seedlings may be at low frequencies initially but may experience strong positive selection over time. Further study is needed to determine the relative importance of these various potential explanations for the geographic pattern in triploidy incidence.

Polyploidy in plants has physiological and structural effects that can have ecological manifestations [18], [66]–[70]. For example, polyploidy is associated with increased cell size and cell water content [66], stomatal size and density [71], and often with changes in growth rates [18], [72]. In woody plants, the increased cell size associated with polyploidy may compromise structural integrity [16], [73]. In other *Populus* species, changes in xylem element sizes alter the risk of cavitation [74], [75]. Thus, physiological and structural differences between aspen cytotypes may result in various ecological advantages and/or vulnerabilities for triploids, and these may vary with the stem age, size, and environment. We posit that triploidy, clonal sizes and stand age structure may be major components of spatial variance in the dramatic aspen mortality patterns recently attributed to climate fluctuations [36], [41], [42].

More generally, our findings suggest that clonality may facilitate the persistence of triploids, and triploidy may facilitate the persistence of large, long-lived clones. Because triploids can serve as a bridge to tetraploid lineages and the rapid evolution of new species [22], [26], the synergy between clonality and triploidy may be an important aspect of speciation in perennial plants.

## Supporting Information

Table S1 **Sample collection sites and summary data.**
(DOCX)
