# Supplementary material for: Widespread Triploidy in Western North American Aspen (Populus tremuloides)
Source: PLoS One. 2012 Oct 31;7(10):e48406. doi: 10.1371/journal.pone.0048406 (PMC3485218; doi:10.1371/journal.pone.0048406)
Supplement: Table S1 — Sample collection sites and summary data. (DOCX) [file pone.0048406.s001.docx]

**Table S1. Sample collection sites and summary data.**

| **Map Code** | **# Samples** | **# Diploids** | **# Triploids** | **# μsats only** | **μsat Set (Table 1)** | **# flow cytometry only** | **# μsats + flow cytometry** | **Proportion Diploids** | **Proportion Triploids** | **Max. distance (km) among sampled ramets** |
| --- | --- | --- | --- | --- | --- | --- | --- | --- | --- | --- |
| AKK | 25 | 15 | 10 | 13 | Set 1 | 0 | 12 | 0.58 | 0.42 | 26 |
| AZ | 48 | 46 | 2 | 48 | Set 7 | 0 | 0 | 0.96 | 0.04 | 7 |
| BCQ | 28 | 28 | 0 | 3 | Set 1 | 10 | 15 | 1.00 | 0.00 | 24 |
| BNF | 27 | 20 | 7 | 0 | Set 1 | 2 | 25 | 0.74 | 0.26 | 30 |
| CANV | 29 | 18 | 9 | 0 | Set 1 | 4 | 25 | 0.62 | 0.31 | 25 |
| CF1 | 11 | 11 | 0 | 11 | Set 1 | 0 | 0 | 1.00 | 0.00 | 33 |
| CF2 | 19 | 18 | 0 | 0 | Set 1 | 0 | 19 | 0.95 | 0.00 | 39 |
| CM | 281 | 88 | 193 | 274 | Sets 5,6 | 0 | 7 | 0.31 | 0.69 | 20 |
| CMNY | 21 | 21 | 0 | 1 | Set 1 | 8 | 12 | 1.00 | 0.00 | 8 |
| CO | 15 | 6 | 9 | 15 | Set 1 | 0 | 0 | 0.40 | 0.60 | 28 |
| COG | 12 | 7 | 4 | 8 | Set 1 | 0 | 4 | 0.58 | 0.33 | 81 |
| CSS | 18 | 11 | 7 | 0 | n/a | 18 | 0 | 0.61 | 0.39 | 30 |
| DLL | 26 | 16 | 10 | 26 | Set 4 | 0 | 0 | 0.62 | 0.38 | 4 |
| DLN | 30 | 30 | 0 | 1 | Set 1 | 16 | 13 | 1.00 | 0.00 | 21 |
| FL | 61 | 52 | 8 | 52 | Sets 1,2 | 0 | 9 | 0.85 | 0.13 | 3 |
| FLFL | 24 | 20 | 4 | 2 | Set 1 | 5 | 17 | 0.83 | 0.17 | 10 |
| GUT | 23 | 10 | 13 | 3 | Set 1 | 8 | 12 | 0.43 | 0.52 | 37 |
| HIN | 25 | 21 | 4 | 2 | Set 1 | 13 | 10 | 0.84 | 0.16 | 34 |
| HSPQ | 26 | 26 | 0 | 3 | Set 1 | 1 | 22 | 1.00 | 0.00 | 23 |
| KFO | 19 | 15 | 4 | 3 | Set 1 | 4 | 12 | 0.79 | 0.21 | 22 |
| LALO | 26 | 24 | 2 | 2 | Set 1 | 10 | 14 | 0.92 | 0.08 | 27 |
| MIUP | 29 | 27 | 1 | 2 | Set 1 | 1 | 26 | 0.93 | 0.03 | 18 |
| MIWI | 68 | 68 | 0 | 68 | Set 1 | 0 | 0 | 1.00 | 0.00 | 24 |
| MN1 | 95 | 94 | 1 | 95 | Set 1 | 0 | 0 | 0.99 | 0.01 | 15 |
| MN2 | 46 | 46 | 0 | 46 | Set 1 | 0 | 0 | 1.00 | 0.00 | 38 |
| MN3 | 23 | 23 | 0 | 13 | Set 1 | 7 | 3 | 1.00 | 0.00 | 8 |
| MTF | 13 | 9 | 4 | 13 | Set 1 | 0 | 0 | 0.69 | 0.31 | 97 |
| MXQ | 19 | 13 | 6 | 19 | Set 1 | 0 | 0 | 0.68 | 0.32 | 4 |
| NBC | 20 | 18 | 2 | 0 | n/a | 20 | 0 | 0.90 | 0.10 | 32 |
| NM | 12 | 11 | 1 | 7 | Set 1 | 5 | 0 | 0.92 | 0.08 | 16 |
| NMC | 16 | 9 | 7 | 16 | Set 1 | 0 | 0 | 0.56 | 0.44 | 75 |
| NMI | 51 | 51 | 0 | 51 | Set 1 | 0 | 0 | 1.00 | 0.00 | 25 |
| NMT | 20 | 12 | 8 | 20 | Set 1 | 0 | 0 | 0.60 | 0.40 | 81 |
| NVW | 15 | 11 | 4 | 15 | Set 1 | 0 | 0 | 0.73 | 0.27 | 48 |
| POTR | 29 | 28 | 1 | 12 | Set 1 | 2 | 15 | 0.97 | 0.03 | 30 |
| SFQ | 28 | 28 | 0 | 8 | Set 1 | 5 | 15 | 1.00 | 0.00 | 26 |
| UAB | 12 | 12 | 0 | 12 | Set 4 | 0 | 0 | 1.00 | 0.00 | 1 |
| UME | 10 | 10 | 0 | 10 | Set 1 | 0 | 0 | 1.00 | 0.00 | 1 |
| USF | 205 | 148 | 57 | 199 | Sets 1,3 | 0 | 6 | 0.72 | 0.28 | 16 |
| WI | 37 | 37 | 0 | 37 | Set 1 | 0 | 0 | 1.00 | 0.00 | 3 |
| WNC | 21 | 21 | 0 | 21 | Set 1 | 0 | 0 | 1.00 | 0.00 | 23 |
| WWA | 19 | 12 | 7 | 16 | Set 1 | 0 | 3 | 0.63 | 0.37 | 16 |
| **Totals** | **1582** |  |  | **1147** |  | **139** | **296** |  |  |  |

Samples with conflicting cytotype information from microsatellites (μsats) and flow cytometry were not tallied in the counts of diploids and triploids by population.
